# Brain-Derived Neurotrophic Factor, Kynurenine Pathway, and Lipid-Profiling Alterations as Potential Animal Welfare Indicators in Dairy Cattle

**DOI:** 10.3390/ani13071167

**Published:** 2023-03-25

**Authors:** Alessandra Favole, Camilla Testori, Stefania Bergagna, Maria Silvia Gennero, Francesco Ingravalle, Barbara Costa, Sara Barresi, Piercarlo Curti, Francesco Barberis, Sandra Ganio, Riccardo Orusa, Elena Vallino Costassa, Elena Berrone, Marco Vernè, Massimo Scaglia, Claudia Palmitessa, Marina Gallo, Carlotta Tessarolo, Sabina Pederiva, Alessio Ferrari, Valentina Lorenzi, Francesca Fusi, Laura Brunelli, Roberta Pastorelli, Giulia Cagnotti, Cristina Casalone, Maria Caramelli, Cristiano Corona

**Affiliations:** 1 Istituto Zooprofilattico Sperimentale del Piemonte, Liguria e Valle d’Aosta, 10154 Turin, Italy; alessandra.favole@izsto.it (A.F.); camilla.testori@izsto.it (C.T.); stefania.bergagna@izsto.it (S.B.); mariasilvia.gennero@izsto.it (M.S.G.); francesco.ingravalle@izsto.it (F.I.); riccardo.orusa@izsto.it (R.O.); claudia.palmitessa@izsto.it (C.P.); marina.gallo@izsto.it (M.G.); carlotta.tessarolo@izsto.it (C.T.); sabina.pederiva@izsto.it (S.P.); alessio.ferrari@izsto.it (A.F.); cristina.casalone@izsto.it (C.C.); maria.caramelli@izsto.it (M.C.); 2 Azienda Sanitaria Locale (ASL) Cuneo (CN), 12100 Cuneo, Italy; barbara.costa@aslcn1.it (B.C.); sara.barresi@aslcn1.it (S.B.); piercarlo.curti@aslcn1.it (P.C.); francesco.barberis@aslcn1.it (F.B.); 3 Azienda USL Valle d’Aosta, Aosta (AO), 11100 Aosta, Italy; sganio@ausl.vda.it; 4 Azienda Sanitaria Locale (ASL) Turin, 10129 Turin, Italy; evallinocostassa@aslto4.piemonte.it (E.V.C.); mverne@aslto3.piemonte.it (M.V.); mscaglia@aslto3.piemonte.it (M.S.); 5 Italian Reference Centre for Animal Welfare (CReNBA), Istituto Zooprofilattico Sperimentale della Lombardia e dell’Emilia Romagna “Bruno Ubertini” (IZSLER), 25124 Brescia, Italy; valentina.lorenzi@izsler.it (V.L.); francesca.fusi@izsler.it (F.F.); 6 Istituto di Ricerche Farmacologiche Mario Negri IRCCS, 20156 Milan, Italy; laura.brunelli@marionegri.it (L.B.); roberta.pastorelli@marionegri.it (R.P.); 7 Department of Veterinary Sciences, University of Turin, 10095 Turin, Italy; giulia.cagnotti@unito.it

**Keywords:** neurobiology, animal welfare, biomarkers, dairy cattle, tie-stall, depression-like state, BDNF, kynurenine pathway, lipidome

## Abstract

**Simple Summary:**

Animal welfare assessment is crucial for farm animal health and productivity. New standardized biomarkers are needed to gain a complete picture of the ethological, physiological, and psychological needs of animals. With this study, we wanted to investigate potential biomarkers for measuring the physical and mental health of dairy cows. Since the stress induced by housing conditions can give rise to multisystem alterations, we compared the effects of three different farming systems. Plasma levels of hematological and inflammatory markers and more than 130 metabolites were investigated by magnetic bead panel multiplex assay and mass spectrometry assay, in addition to brain and plasma levels of brain-derived neurotrophic factor (BDNF), a neurotrophin involved in neuroplasticity and synaptogenesis, and indoleamine 2,3-dioxygenase, an enzyme whose dysregulated activity in humans has been correlated with mood disorders. Our findings suggest that because BDNF level, kynurenine pathway, and lipid-profiling alterations may modulate a depression-like state in tie-stall cattle, they may be potential biomarkers for monitoring dairy cattle welfare.

**Abstract:**

Complete animal welfare evaluation in intensive farming is challenging. With this study, we investigate new biomarkers for animal physical and mental health by comparing plasma expression of biochemical indicators in dairy cows reared in three different systems: (A) semi-intensive free-stall, (B) non-intensive tie-stall, and (C) intensive free-stall. Additionally, protein levels of mature brain-derived neurotrophic factor (mBDNF) and its precursor form (proBDNF) and indoleamine 2,3-dioxygenase (IDO1) specific activity were evaluated in brain samples collected from 12 cattle culled between 73 and 138 months of age. Alterations in plasma lipid composition and in the kynurenine pathway of tryptophan metabolism were observed in the tie-stall-reared animals. The total plasma BDNF concentration was higher in tie-stall group compared to the two free-housing groups. Brain analysis of the tie-stall animals revealed a different mBDNF/proBDNF ratio, with a higher level of proBDNF (*p* < 0.001). Our data are similar to previous studies on animal models of depression, which reported that inhibition of the conversion of proBDNF in its mature form and/or elevated peripheral kynurenine pathway activation may underlie cerebral biochemical changes and induce depressive-like state behavior in animals.

## 1. Introduction

Improving farm animal welfare status is a critical need since there is sufficient scientific knowledge indicating the capacity of animals to experience pain, distress, and suffering. Animal wellbeing is closely connected with animal health and food safety, while the ethics of intensive farm practices have been increasingly called into question by public opinion [1]. Animal welfare has been traditionally framed by Brambell’s five freedoms [2]: wellbeing is expected to occur if animals are free: (1) from thirst, hunger, and malnutrition, (2) from thermal and physical discomfort, (3) from pain, injury, and disease, (4) to express their natural behavioral repertoire, and (5) from fear and distress. Research has shown, however, that the absence of suffering does not necessarily mean a good state of welfare; therefore, cognitive and emotional aspects and mental health need to be evaluated as well [3,4].

Internal and external inputs are processed in the central nervous system (CNS), where innate physiological and emotional responses are transmitted via efferent pathways and manifested in overt behavioral patterns [5]. Furthermore, mental suffering (phobia, anxiety, compulsion, depression) and psychological stress (e.g., social competition and intraspecific conflict) can compromise the immune system of farm animals and their physical health [6]. While animal-based measures such as those introduced by the Welfare Quality^®^ Project [7] are useful for studying the physical and mental welfare of farm animals, they are recorded during physical examinations and behavioral observation. A more complete and objective evaluation of animal welfare could be gained with specific and measurable animal-based biomarkers of mental health and emotional stress.

Brain-derived neurotrophic factor (BDNF) is the most abundant growth factor in the CNS [8]. It is essential for CNS development and neuronal plasticity. The precursor form (proBDNF) promotes cell death and growth cone retraction, dendritic spine shrinkage, and long-term depression, whereas the mature form (mBDNF), cleaved from proBDNF, promotes neuronal survival, spine protrusion, and long-term potentiation [9]. Because of its role in neuroplasticity and synaptogenesis, BDNF has been widely implicated in neurodevelopmental and psychiatric diseases. Low levels in humans have been associated with major depression disorder (MDD), schizophrenia, addiction, post-traumatic stress disorder, and suicidal behavior [9,10,11]. Furthermore, the altered conversion of proBDNF to mBDNF in mice was found to be associated with depressive-like behavior and impaired nest building [12] and chronic stress associated with low hippocampal mRNA and protein BDNF levels [9]. Serum BDNF levels have been associated with environmental enrichment in pigs: higher levels of mBDNF were found in animals housed in an enriched environment compared to those kept in a barren setting [8]. In summary, the presence of BDNF in the brain and the peripheral tissues make it an interesting candidate as a biomarker for animal welfare evaluation [1,10,13].

Moreover, the kynurenine pathway could be a useful molecular indicator for animal wellbeing assessment by virtue of its role in regulating the chronic stress response [14]. An essential amino acid, tryptophan (TRP), and its metabolism play a critical role in the pathophysiology of depressive disorders [15]. Nearly 95% of TRP is metabolized via the kynurenine pathway in the CNS and the periphery by indoleamine 2,3-dioxygenase (IDO1), whereas only 1% is converted to serotonin and melatonin via the serotonin pathway. Activation of IDO1 and increased levels have been correlated with the shunting of the TRP metabolism, from 5-hydroxytryptamine (5-HT) production to kynurenine (KYN) production, which is involved in the pathophysiology of many mood disorders and in suicidal ideation in both adults and adolescents [14,16]. Furthermore, stress may induce an imbalance in KYN-pathway metabolites downstream, and the KYN/TRP ratio may function as a neuromediator between stress and behavioral changes in addiction disorders [17]. Conversely, KYN accumulation can be suppressed by activating kynurenine clearance in exercised skeletal muscle in a mouse model of stress-induced depression [18]. Metabolites of the TRP pathway, investigated as animal welfare indicators in hens subjected to social disruption, showed increased TRP levels and KYN/TRP ratios in the hens displaying feather pecking behavior [19].

Recently, lipidomic approaches by liquid chromatography coupled to MS (LC-MS) and tandem MS (LC-MS/MS) have studied the lipidome from the brain and its subregions [20,21] in mice [22], as well as body fluids (e.g., plasma, serum, cerebrospinal fluid), to identify characteristic markers for the diagnosis of brain conditions and disorders such as MDD [23]. Previous studies have reported changes in the lipidome of the brain or the plasma of animal models of disease as well as in plasma and serum from patients with MDD [24].

To date, animal welfare in intensive animal farming has been assessed mainly using resource-based measures and animal-based indicators of health/stress parameters. A complete picture of the ethological, physiological, and psychological needs of farm animals could be gained with the use of standardized animal-based biomarkers. With this study, we investigate biomarkers that could provide a clearer view of the mental state of animals and their ability to cope with their environment. To do this, we applied a multi-method quantitative approach to three different intensive dairy farming models.

## 2. Materials and Methods

### 2.1. Animals

The study was conducted on 41 dairy cows, each reared in different housing conditions and/or milk productivity levels: Farm A—free-stall housing (*n* = 13) with medium or semi-intensive milk production (12–40 kg/cow per day); Farm B—tie-stall housing (*n* = 13 cows tethered at the neck to their stall) with low or non-intensive milk production (≤12.5 kg/cow per day); Farm C—free-stall (*n* = 15) with high or intensive production (>40 kg/cow per day). The cows in the tie-stall housing group were always kept tied and milked in their stall, while those reared in free-stall housing were kept for a year in the cubicle house and then allowed to roam in a yard outside the stalls. Table 1 presents the general data (age, breed, etc.) of the animals. The herd health status was officially free of bovine tuberculosis, bovine brucellosis, and enzootic bovine leukosis. The animals had been vaccinated for infectious bovine rhinotracheitis virus and were serologically negative for *Mycobacterium avium* spp. *paratuberculosis*.

### 2.2. On-Farm Animal Welfare Assessment

A trained veterinarian applied two different protocols to assess animal welfare: one for the tie-stall system and the other for the free-stall system. The protocols were developed by the Italian National Reference Centre for Animal Welfare (CReNBA), Istituto Zooprofilattico Sperimentale della Lombardia e dell’Emilia Romagna (IZSLER). They are currently posted on the ClassyFarm platform of the Italian Ministry of Health for monitoring and benchmarking animal welfare on farms [25,26].

The protocols list resource- and animal-based indicators in a multiple-choice item checklist. The indicators refer to the minimum legal requirements for the protection of farm animals (Council Directive 98/58/EC, transposed by Italian law Decreto Legislativo no. 146/2001; Council Directive 2008/119/CE, transposed by Italian law Decreto Legislativo no. 126/2011; European Food Safety Authority recommendations for dairy cow welfare (EFSA, 2012); and the Welfare Quality^®^ protocol for dairy cattle (Welfare Quality Consortium, 2009)) [27].

The welfare assessment protocol for tie-stall farms lists 58 indicators, while the protocol for free-stall farms lists 70 indicators. The indicators are grouped by section: Section A—farm management and staff training, Section B—housing and equipment, and Section C—animal-based measures (ABMs) [25]. Each indicator has two or three well-defined risk levels: level 1 indicates a high risk or poor status of the indicator, level 2 indicates a medium risk, and level 3 indicates a low risk or better status of the indicator [27,28]. Each indicator level differs in weight according to its potential impact on dairy cow welfare [27]. The weight is used for calculating the section scores and the total welfare score [25].

The section scores are calculated by summing the scores of the indicators in each section. The total welfare score is calculated with 50% from Sections A and B and the other 50% from Section C. Section and total welfare scores are expressed in percentages, from 0 to 100, where 0 denotes a poor section score or poor animal welfare status and 100 an excellent section score or optimal animal welfare status [25].

### 2.3. Plasma Collection, Biochemical Profile, and Health Status

The animals underwent clinical examination and blood sampling by the veterinarian responsible for the herd. Blood samples were collected in the framework of obligatory and voluntary health programs (e.g., certified disease-free programs in the European Union) carried out by the Italian Animal Health Service. To minimize variability in analyte concentration, all blood samples were taken at the same time of the day from the subcaudal coccygeal vein using tubes with anticoagulant (EDTA K3), and centrifuged at 2500× *g* for 15 min. The plasma fraction was transferred and stored at −80 °C until assay. Blood count was performed within 24 h of collection on a Melet Schloesing^®^-MS4 instrument (Melet Schloesing Laboratoires, Osny, France). A general state of health was determined by measuring hepatic (alkaline phosphatase (ALP), aspartate aminotransferase (AST) and alanine aminotransferase (ALT)), renal (creatinine (CREA), and urea (UR)), and lipidic (triglyceride (TG), and total cholesterol (T-Chol)) profiles on an automated system photometer (I-Lab Aries Chemical Analyzer—Instrumentation Laboratory, Bedford, MA, USA). Lysozyme and serum bactericidal activity were determined as described elsewhere [29]. Analysis to quantify plasma C-reactive protein (CRP) was carried out using commercial kits according to the manufacturer’s instructions and standard procedures. A sandwich ELISA kit (Bovine C-Reactive Protein ELISA Kit, Bioassay Technology Laboratory, Shanghai, China) was used.

### 2.4. MILLIPLEX^®^ Bovine Cytokine/Chemokine Magnetic Bead Panel Multiplex Assay

Plasma harvested from whole blood was screened on a MILLIPLEX^®^ Bovine Cytokine/Chemokine 15-plex kit (BCYT1- 33 K; EMD Millipore, Billerica, MA, USA) utilizing antibodies to bovine IFN-γ, interleukin (IL)-1α, IL-1β, IL-4, IL-6, IL-8, IL-10, IL-17A, macrophage inflammatory protein (MIP)-1α, IL-36 receptor antagonist (Ra), IP-10, macrophage chemo-attractant protein (MCP)-1, MIP-1β, tumor necrosis factor (TNF)-α, and vascular endothelial growth factor (VEGF)-A according to the manufacturer’s instructions and as reported by Smith et al. [30]. Briefly, plasma samples were diluted 1:2 in assay buffer before adding 25 μL of standards, quality controls, and samples to the plate in duplicate, followed by 25 μL of magnetic beads. The plate was sealed, covered with foil, and incubated for 2 h on a plate shaker at room temperature (RT). The plate was washed three times, and 25 μL of detection antibody was added to each well. After incubating the plate for 1 h at RT, 25 μL of streptavidin–phycoerythrin (PE) was added per well. The plate was sealed, covered, and incubated for 30 min at RT. The plate underwent a final series of washes, and then, 150 μL of drive fluid was added. Marker concentrations were measured on a Bio-Plex 200^®^ plate reader (Bio-Rad, Hercules, CA, USA). Quality control values for each marker were consistently within the range stated by the manufacturer.

### 2.5. Target Metabolomics Analysis

A targeted quantitative approach using a combined direct flow injection and liquid chromatography (LC) tandem mass spectrometry (LC-MS/MS) assay (AbsoluteIDQ 180 kit, Biocrates Life Science, Innsbruck, Austria) was applied for metabolomics analysis of the EDTA-plasma samples stored at −80 °C. This method allows the simultaneous absolute quantification of 186 metabolites (40 amino acids and biogenic amines, including serotonin and kynurenine, 40 acylcarnitines, 90 glycerophospholipids, 15 sphingomyelins and 1 monosaccharide).

The plasma samples (10 µL) were processed following the manufacturer’s instructions and analyzed on a triple-quadrupole mass spectrometer (AB SCIEX triple-quad 5500) operating in the multiple reaction monitoring (MRM-MS) mode. The assay is based on PITC (phenylisothiocyanate)-derivatization in the presence of internal standards for the analysis of amino acids and biogenic amines resolved and quantified by LC-MS/MS using scheduled MRMs. Subsequent flow injection analysis tandem mass spectrometry (FIA-MS/MS) was performed to analyze acylcarnitines, glycerophospholipids, and hexose. MRM detection was used for quantification by applying spectra parsing algorithms integrated into the MetIQ software (Biocrates Life Science). Concentrations were calculated and evaluated by comparing analytes measured in a defined extracted ion count section to those of specific, labeled internal standards or non-labeled ones provided with the kit. The measurements were made in a 96-well format. Seven calibration standards, four quality control samples, three zero samples (PBS), and one blank (solvents) were integrated into the plate. Triplicate analysis of duplicates of plasma samples yielded an average coefficient of variance (CV%) below 0.16 for the metabolites, the lowest being for amino acids (CV% 0.1) and the highest for sphingomyelins (CV% 0.23). To ensure data quality and robust statistical analysis, the following filtering criteria were applied: metabolites measured with more than 20% missing data (no detectable peak) were excluded from further data analysis; metabolites for which the plasma concentration was below the limit of detection (<LOD) in at least ≥50% of the analyzed samples were excluded. The KT ratio in plasma was calculated as plasma KYN concentration to plasma TRP concentration.

### 2.6. Determination of Plasma BDNF

Total plasma BDNF was measured using an ELISA kit (Bovine BDNF PicoKine ELISA kit, Boster Biological Technology, Pleasanton, CA, USA; standard curve range 31.2–2000 pg/mL; sensitivity: <2 pg/mL).

### 2.7. Slaughterhouse Procedure, Brain Collection, and Western Blot Analysis of proBDNF and mBDNF Expression in CNS

Cows selected for brain sampling (Table 1) were regularly slaughtered in a certified slaughterhouse following animal protection legislation at the time of killing (Council Regulation (EC) no. 1099/2009 and no. 1/2005). The three stages were: preslaughter handling, stunning, and slaughtering. During lairage time, the animals were housed in a holding pen designed to allow them proper space to stand up or lie down and to ensure free access to water. An ante mortem inspection was performed, and the animals were driven to the stunning area in a quiet and orderly manner. The stunning method was mechanical, using a penetrative captive bolt pistol, and performed by properly trained and competent personnel.

Brain areas (hippocampus, cerebellum, thalamus, frontal and occipital cortex) were sampled and homogenized at 20% weight/volume (*w*/*v*) in RIPA (Merck, Darmstadt, Germany) lysis buffer with an added cocktail of protease inhibitors (cOmplete™ protease inhibitor cocktail tablets™, Roche Diagnostics, Monza, Italy). Protein quantification was performed with a Qubit Protein Assay Kit™ (Thermo Fisher Scientific, Waltham, MA, USA); 15 μg of each sample were loaded onto Mini Protean TGX ™ gel (4–20%, Bio-Rad) and separated by electrophoresis for 50 min at 150 V using a Miniprotean II™ (Bio-Rad) chamber. The PVDF (Bio-Rad) membrane blot was obtained at a voltage of 25 V for 5 min on a semi-dry Trans-Blot-Turbo™ (Bio-Rad) transfer system according to the manufacturer’s protocol for low MW. The PVDF membranes were saturated for 2 h at RT in 0.2% I-BLOCK ™ saline solution (Thermo Fisher Scientific). Protein was detected with anti-BDNF ab108319 monoclonal antibody (1: 5000, AbCam Cambridge, UK), which recognizes the mature form of BDNF (mBDNF, 15 KDa), its precursors proBDNF (32 KDa), and preproBDNF (40 KDa). The expression of bovine β-tubulin was used as a housekeeping gene and detected with monoclonal antibody MAB1637 (1:5000, Millipore, Burlington, MA, USA). The membranes were incubated with 1 mL of the combined 1:1 solution of the Clarity ™ Western ECL Substrate detection kit (Bio-Rad) and acquired in chemiluminescence using the ChemiDoc™ Touch image acquisition system (Bio-Rad). Semi-quantitative analysis of protein expression by Western blot of the mature form of BDNF (mBDNF) and its precursor (proBDNF) was performed. ImageLab™ (Bio-Rad) image analysis software was used to quantify signal intensity.

### 2.8. CNS Indolamine 2,3-Dioxygenase (IDO1) Enzymatic Activity

Endogenous enzymatic activity of IDO1 was quantified using a fluorimetric assay (#K972-100, Biovision, Milpitas, CA, USA) on the frontal cortical and the thalamus. Briefly, the brain tissues were homogenized at 20% *w*/*v* in lysis buffer and prepared according to the manufacturer’s instructions. Protein quantification was performed with a Qubit™ Protein Assay Kit (Thermo Fisher Scientific). IDO1 *specific activity* was calculated according to the formula:

*IDO1 Specific Activity* = *B*/*T* × *P* = pmole/min/mg = µU/mg

where *B* is the quantity of *N*-formylquinurenine (NFK) produced, calculated from the standard curve (in pmole), *T* is the reaction time (in min), and *P* is the amount of protein in the well (in mg). Linear regression fit was based on the mean blank corrected in pmole (400-10/482-10). A unit of IDO1 activity was defined as the amount of enzyme that generates 1 µmole of *N*-formylquinurenine detected (NFK) per minute by the oxidative metabolism of 1 µmole l-tryptophan at 37 °C (standard curve range 0–2000 pmole NFK; sensitivity 0.2 mU of IDO1 activity or 200 pmole NFK).

### 2.9. Statistical Analysis

Separate statistical analyzes were conducted using StataCorp. 2021 (Stata Statistical Software: Release 17. College Station, TX USA: StataCorp LLC) software. This was done to determine statistically significant differences between the three farms. Descriptive statistics (mean, median, minimum, maximum, standard deviation) of hematological, blood biochemical, and immunological profiles were estimated for each farm; a non-parametric local–linear and local–constant kernel regression model [31,32] was fitted for each indicator to find differences in the expression of metabolomic indicators between the farms.

Metabolite concentration (µM) underwent multivariate data analysis (SIMCA-P13 software package, Umetrics, Sartorius, Göttingen, Germany). Metabolite levels were Pareto-scaled with mean centering and analyzed by unsupervised principal component analysis (PCA) and supervised partial least squares discriminant analysis (PLS-DA) to maximize class discrimination. Score plots generated by PCA and PLS-DA were used to visualize clustering. Variable importance in the project (VIP) score was used to estimate the importance of each variable in the projection in the PLS model for extracting the metabolites with the highest magnitude and the highest reliability for discriminating between groups.

Descriptive statistics for plasma BDNF levels were produced for each farm; differences between farms were tested using the Kruskal–Wallis test and the Wilcoxon rank-sum test [33]. To determine the effect of housing systems and milk productivity on BDNF and the kynurenine pathway in the CNS, descriptive statistics and box plots were produced for each farm, and a repeated-measures ANOVA with Bonferroni-adjusted significance tests for pairwise comparisons was fitted on the BDNF ratio.

## 3. Results

### 3.1. On-Farm Animal Welfare Assessment

Table 2 presents the results of the animal welfare assessment. None of the farms showed criticalities for minimal legislative requirements. Farms A (free-stall system with medium daily milk production) and C (free-stall system with high daily milk production) had a total animal welfare score of 65.5% and 70.5%, respectively, whereas Farm B (tied-stall system with low daily milk production) had the lowest animal welfare score (48.9%). Farm A had the highest ABM score (76.1%), followed by Farms C (67.4%) and B (54.5%).

### 3.2. General Health Status

Physical examination and history taking by the veterinarian responsible for herd health revealed no clinical signs of disease. Table 3 and Table 4 present descriptive statistics, with estimated means and confidence intervals (CI) for hematological and blood biochemical parameters for Farms A, B, and C, respectively. A comparison of the measurements with reference laboratory values for healthy cows in full/late lactation [34,35,36,37] showed a good general state of health in all dairy cattle. The non-parametric local–linear and local–constant kernel regression model showed no significant difference in blood biochemical parameters between the farms.

Plasma samples were analyzed for the concentration of 15 cytokines/chemokines using a multiplex cytokine bead array assay. Analytes for which the plasma concentration was below the limit of detection (<LOD) in at least ≥50% of the samples were excluded. Table 5 presents the descriptive statistics of the estimated mean and range for each analyte for the animals from Farms A, B, and C. Variance analysis (Kruskal–Wallis test and Wilcoxon rank-sum test for pairwise comparison, KW) revealed no difference in the plasma levels of 10 out of 12 cytokines and chemokines between the groups. A slight albeit statistically significant increase in anti-inflammatory cytokines IL-10 (*p* < 0.01) and IL-36 receptor antagonists (*p* < 0.05), respectively, was observed in the animals from farms C and B.

### 3.3. Target Metabolomics Analysis

We applied mass-spectrometry-based quantitative metabolomic profiling to identify and quantify lipids, amino acids, biogenic amines, and acylcarnitines in plasma (*n* = 10/group). In total, 135 metabolites were identified: 1 hexose, 21 amino acids, 13 biogenic amines, 3 acylcarnitines, 15 sphingomyelins species (SM), 71 phosphatidylcholines species (PC), and 11 LysoPC species. Concentrations (as µM) are reported in Table S1 in the Supplementary Material.

**Figure 1:**
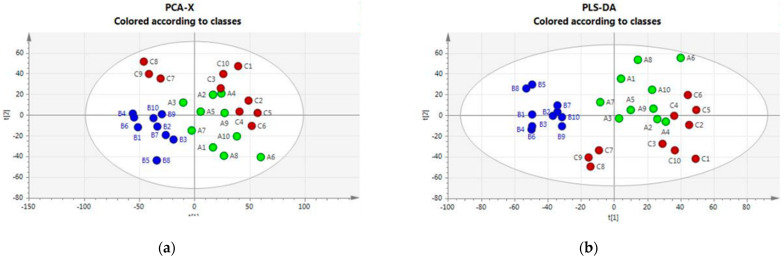
(**a**) PCA score plots and loading plots. Green class: Housing-A; Blu Class: Housing-B; Red class: Housing-C. (**b**) PLS-DA score plots and loading plots. Green class: Housing-A; Blu Class: Housing-B; Red class: Housing-C.

An initial broad metabolic comparison between the three groups was made using multivariate data analysis (MVDA) with unsupervised (principal component analysis, PCA) and supervised (partial least squares discriminant, PLS-DA) analysis to allow for cattle–housing clustering. Figure 1 (Panels a and b) shows that the PCA and PLS-DA score plots indicate an impact of the housing system on the plasma metabolomics profile. Housing systems on Farms A and C were separated from the system on Farm B; some outliers were observed mainly for Farm B.

The VIP scores reflecting the importance of metabolites in the PLS-DA model are reported in Table S2 in the Supplementary Material. They highlight the relevance of unsaturated long-chain phosphatidylcholine species and some amino acids in driving the observed class separation.

All metabolite groups were compared by variance analysis (Kruskal–Wallis and Wilcoxon rank-sum test for pairwise comparison) and non-parametric local–linear and local–constant kernel regression models. Both analyses showed significant differences (*p* < 0.001) between the housing systems for some amino acid and long-chain PC species expression in plasma (Table 6). The average concentration of PC and SM species recorded for Farm B (lysoPCaC18:2, PCaaC32:3, PCaaC36:1, PCaaC36:3, PCaaC36:4, PCaaC38:3, SMC16:0) was 10-fold less than for Farm A or Farm C, while PCaaC34:2 and PCaaC36:2 were 100-fold less. Compared to Farms A and C, the measurements recorded for Farm B showed the lowest average serotonin level (B = 1.61 µM; CI 95% [1.06–2.96]) and the highest kynurenine level (12.40 µM; CI 95% (9.82–14.18)) and KYN/TRP ratio (0.36; CI 95% (0.27–0.53)). Significant differences were also observed for kynurenine, TRP, and the KYN/TRP ratio between Farms B and A (*p* < 0.05) and Farms B and C (*p* < 0.05). Table 6 presents significant test results.

### 3.4. Plasma BDNF

**Figure 2:**
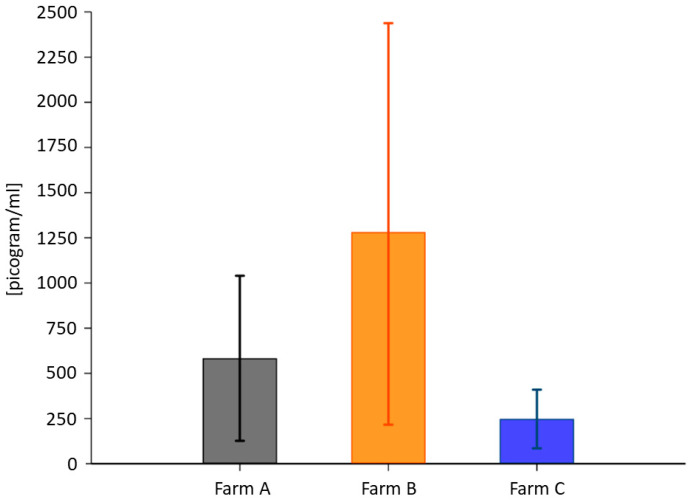
Average plasma BDNF concentration expressed as pg/mL. Error bars denote confidence intervals (>95%).

ELISA was run to measure plasma BDNF. The total BDNF in the cows from Farm B (1281.63 ± 1763 pg/mL) was higher than in those from Farm A (583.37 ± 755.57 pg/mL) and Farm C (233.93 ± 253.67 pg/mL) (Figure 2). Table 7 presents the descriptive statistics for the average value. The measurements from the three groups were compared with the Kruskal–Wallis test (*p* = 0.0022, df2). The Wilcoxon rank-sum test for pairwise comparison showed no significant differences in BDNF levels for Farm A vs. Farm B (*p* = 0.1178; df1) but some differences for Farm A vs. Farm C (*p* = 0.0092; df1) and Farm B vs. Farm C (*p* = 0.0026; df1).

**Figure 3:**
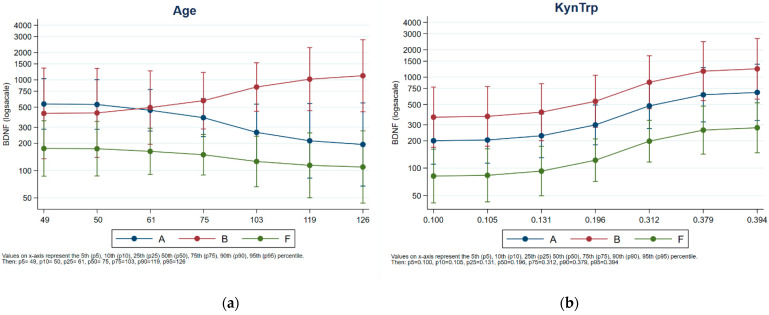
Effect of KYN/TRP ratio (**a**) and ages (**b**) on plasma BDNF levels. Blu Class: Housing-A; Red class: Housing-B. Green class: Housing-C.

Next, ANCOVA was carried out to evaluate the effect of variables (age, kynurenine level, TRP, and KYN/TRP ratio) on plasma BDNF. Significant effects of KYN/TRP (*p* = 0.0122) and KYN (*p* = 0.0362) were observed on the modulation of BDNF, regardless of the farm. No significant effect of age on BDNF level was observed (*p* = 0.6962) for the animals >75 months of age in tie-stall housing; they tended to have higher levels of BDNF than the animals in free-stall housing, whose levels tended to decrease with increasing age. Figure 3 presents a comparison. Animal age was evenly distributed across herds (Figure S1 in the Supplementary Material).

### 3.5. Effect of Housing System and Milk Productivity on BDNF and Kynurenine Pathway in the CNS

**Figure 4:**
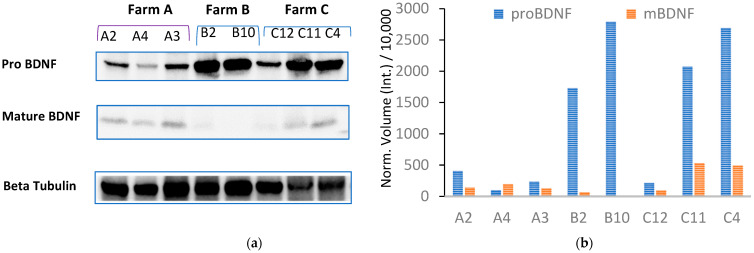
(**a**) Western blot analysis of hippocampal homogenate from Farm A cows (lanes A1, A4, A3), Farm B cows (lanes B2 and B10), and Farm C cows (lanes C12, C11, and C4) was performed to measure the expression of mBDNF and pro-BDNF. (**b**) Expression of proBDNF and mBDNF is shown as normalized volume against bovine β-tubulin used as a housekeeping gene.

To investigate the effect of housing systems and milk productivity on the BDNF pathway in the CNS, we measured the expression of the mature form of BDNF (mBDNF) and its precursor (proBDNF) by repeated Western blot analysis (Figure 4; Figures S2 and S3 in the Supplementary Material) of the hippocampus collected from 12 dairy cows (A, *n* = 4; B, *n* = 3; C, *n* = 5, Table 1), previously assayed for total plasma BDNF. Individual proBDNF and mBDNF volumes were normalized against bovine β-tubulin expression prior to analysis. Figure 2b shows very low or no expression of mBDNF combined with high proBDNF expression detected in cattle B2 and B10, reared in permanent tethering.

**Figure 5:**
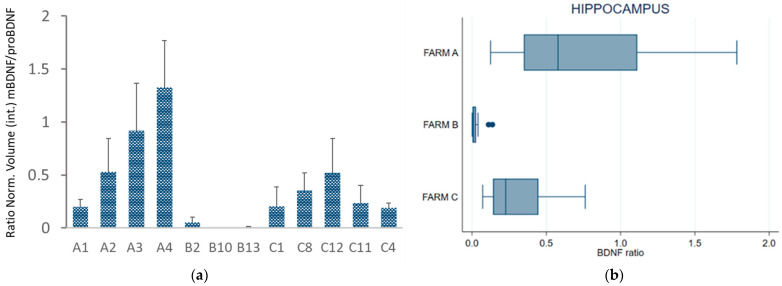
(**a**) BDNF ratio (mBDNF/proBDNF) of normalized volume calculated in the hippocampus; (**b**) box plot of the mBDNF/proBDNF ratio in the hippocampus of the cows grouped by farm. The error bars indicate standard deviation. Individual cows are indicated as: A1, A2, A3, A4 for Farm A; B2, B10, B13 for Farm B; C1, C4, C8, C11, C12 for Farm C.

Figure 5a,b presents, respectively, the BDNF ratio (mBDNF/proBDNF) for each subject and a box plot of the average BDNF ratio measured in the three groups (farms). Western blot of the hippocampus showed a lower BDNF ratio for the cows kept in permanent tethering (farm B) than for those from the other two farms.

Repeated-measures ANOVA and Bonferroni-adjusted significance test for pairwise comparison revealed statistically significant differences between the cows from Farms A and B (ratio −1.74, se 0.27, z −6.46, *p* < 0.001) and between the cows from Farms C and B (ratio +1.37, se 0.26, z 5.26, *p* < 0.001), whereas no statistically significant differences were found between Farms A and C (ratio −0.37, se 0.22, z −1.72, *p* = 0.258).

Endogenous IDO1 activity was quantified by enzymatic fluorimetric assay on brain samples (thalamus and frontal cortex) collected from 12 culled dairy cows and previously assayed. The measures fitted the standard curve range and the detection limits of the kit applied, with an enzymatic activity range specific for IDO1 between 0.1–0.7 µU/mg.

**Figure 6:**
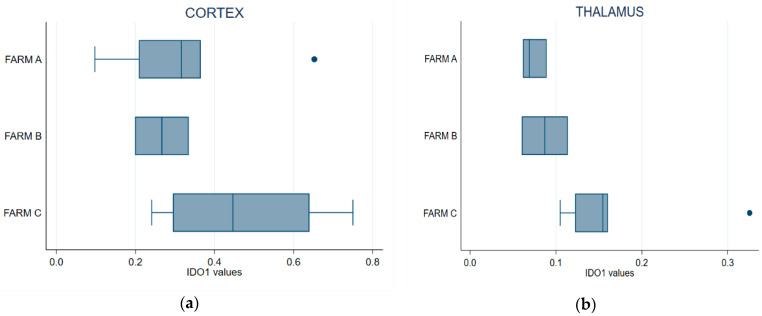
Box plot of IDO1 endogenous enzyme activity in the thalamus (**a**) and the cerebral cortex (**b**) samples. Error bars denote standard deviation.

Figure 6a shows that IDO1 expressed at the frontal cortex of the cows from Farm C was higher than in those from the other two farms. The Kruskal–Wallis test showed that cortical IDO1 levels did not differ between the farms (*p* = 0.2412). IDO1 specific activity in the thalamus was higher in the cows from Farm C (Figure 6b); the difference was statistically significant, indicating that this farm differed from Farm A (Kruskal–Wallis, *p* = 0.0376). Pairwise comparison with the Wilcoxon test showed a statistically significant difference between Farms A and C (*p* = 0.0201) but not between Farms A and B (*p* = 1); a comparison of Farm B vs. Farm C suggested a difference that was not formally supported at 95% (*p* = 0.0956) but might approach significance with a larger sample size.

IDO1 specific activity in the thalamus was related to the level in the frontal cortex. Pearson’s correlation coefficient was *r* = 0.7832 (*p* = 0.0044), while Spearman’s rank correlation coefficient was *r* = 0.7727 (*p* = 0.0053) (Figure S4 in the Supplementary Material).

## 4. Discussion

Interdisciplinary studies have addressed the challenges to animal welfare in industrial livestock production (e.g., confinement, social isolation, overcrowding, lack of natural behavior, stress during transport) [38]; however, animal welfare in intensive farming poses problems that are difficult to define and measure. To date, animal welfare evaluation in intensive farming has relied mainly on resource-based indicators and animal-health-based parameters [27].

Intensive dairy cow farming has undergone modernization to improve biosecurity, animal welfare, and productivity, yet many problems remain. For example, intensive indoor dairy farms can house many hundreds, sometimes thousands, of cows confined for most, if not all, of their lives. To increase milk production, the cows are milked three times a day for up to five years, with the risk of developing lameness and udder infections [39].

Here, we compare the expression of biochemical indicators in cows reared in three different farm management and housing systems (semi-intensive free-stall, intensive free-stall, and non-intensive tie-stall). Our aim was to investigate potential animal-based biomarkers for measuring animal physical and mental health. To do this, we quantified immunological, metabolomical, and neurobiological analytes and identified differences in the expression of BDNF, kynurenines, serotonin, tryptophan, and other metabolites such as amino acids and lipids within the phosphatidylcoline (PC aa) classes.

The cows housed in the tethered tie-stall system were recorded to have the lowest total scores for all assessment sections of the on-farm welfare protocol, including Section C (ABMs), but higher concentrations of total plasma BDNF compared to the cows housed in the free-stall systems. Given that blood BDNF concentrations reflect brain-tissue BDNF levels across species [40], we next quantified the expression of proBDNF and mBDNF in the hippocampus of late-career dairy cows, previously assayed for total plasma BDNF. The results showed an impaired BDNF ratio in the cows housed in permanent tethering due to the increased expression of proBDNF protein in the hippocampus and a downregulated expression of mBDNF. Our hypothesis was that the higher plasma BDNF concentration detected in the tie-stall animals was probably due to the reduced conversion of proBDNF to mBDNF and the resulting accumulation of proBDNF in the CNS. This effect might increase with age: the plasma BDNF levels tended to be higher in the tie-stall animals >75 months of age than in the free-stall animals, in which the levels were observed to decrease with increasing age.

Previous studies have reported that an mBDNF/proBDNF imbalance may induce anxiety or depression [41] and demonstrated an altered proteolytic cleavage of proBDNF to mBDNF in patients with major depressive disorder and in animal models of depression [12]. Furthermore, early enriched environments are known to induce an increased conversion of proBDNF to mBDNF in rat hippocampus and pig serum, respectively [8,42]. Additionally, higher physical activity levels have been correlated with a rise in mBDNF levels in plasma and the brain [43]. In contrast, the lower mBDNF/proBDNF ratio and the increased plasma BDNF concentration we observed in the cows housed in the tie-stall system suggest an effect of this housing system on the reduced conversion of proBDNF to mBDNF, which might induce a depressive-like state in dairy cattle.

Comparison of the data on the kynurenine pathway (KP) in the tie-stall animals versus the free-stall animals and the welfare protocol evaluation and the BDNF results showed lower serotonin levels and increased kynurenine (KYN) in the cows with the lowest ABM scores and the highest proBDNF levels. The metabolic enzyme indoleamine 2,3-dioxygenase (IDO1) has been suggested as a biological mediator of inflammation in the psychopathology of depression, with increased KYN in the tryptophan (TRP) metabolic pathway resulting in reduced serotonin [44]. The KYN to TRP concentration (KT ratio) is, in fact, associated with IDO1 activity [45].

Our results showing a higher KT ratio and increased plasma kynurenine concentration in the tie-stall animals are shared by previous studies that have demonstrated a key role of the peripheral kynurenine pathway in modulating anxiety- and depression-like behaviors in mice [46]. More recently, a growing body of evidence has strengthened the hypothesis for the involvement of KP in the pathophysiology of depression and other stress-related disorders. Indeed, the KYN/TRP ratio was found to be increased in the blood of patients suffering from MDD [47]. Consistent with previous work [46], our results suggest that elevated peripheral KP activation may underlie cerebral biochemical changes and consequent involvement in the induction of depressive-like behavior in the animals housed in the tie-stall system.

No association between milk production, BDNF levels, and kynurenine pathways in cattle has been reported except by studies showing a positive effect of BDNF on fat and lactose synthesis in milk and elevated IDO activity in the serum of dairy cows with mastitis, respectively [48,49]. Here, we compared two free-stall systems with intensive (>40 kg/cow per day) or semi-intensive (12–40 kg/cow per day) daily milk production to evaluate the effect of daily milk production on BDNF expression and KP activation.

We observed the lowest levels of plasma BDNF in the animals with the highest level of milk production but no difference in the mBDNF/proBDNF ratio in the CNS. From this observation, it appears that high dairy production does not induce a reduced conversion of proBDNF to mBDNF and the accumulation and peripheral release of proBDNF, which was observed in the tie-stall animals. Conversely, IDO1 activity in the thalamus samples was higher in the animals with high milk production (housing system C) than in those with medium milk production (housing system A). While peripheral KP activation was lowest at the time of blood withdrawal in the cows kept in housing system C, the prolonged stress conditions due to daily intensive milk production (>40 kg/cow per day) and the stronger social competition may have been the cause of the increased IDO activity detected in the CNS, suggestive of a state of psychological stress induced in these cows [50]. Recently, disturbances in lipid profiling have also been recognized in MDD, in animal models of MDD, and in patients with depression [24,51]. The typical glycerophospholipids found in mammalian membranes are phosphatidylcolines (PCs) [52]. We found alterations in lipid levels, mainly among those within the PC classes. For instance, PC aa C34:2, PC aa C36:2, and PC aa C36:4 were downregulated in the plasma of the tie-stall animals compared to the free-stall animals. Chan and colleagues found changes in plasma lipid profiles, with lower levels of PC 36:4 in a rat model of depression [53]. Changes in plasma phosphatidylcholine and sphingomyelin concentrations were also associated with depression and anxiety symptoms in a family-based lipidomics study [54]. Furthermore, plasma upregulation of different PC species has been found after antidepressant treatment in rodents and humans [55,56]. Although plasma lipid levels are known to be influenced by both diet and lactation stage, the variability we observed in the expression of some PC classes between the tie-stall animals and the free-stall animals cannot be accounted for solely by individual variations in diet or stages of lactation alone. Furthermore, all the animals were in the same lactation cycle, and the diet formulas were similar on all three farms.

These findings suggest that alterations in lipid plasma profiles, together with impaired proBDNF expression and peripheral KP activation, may have a role in the induction of depression-like state behavior in tie-stall-reared cattle and may be a potential biomarker for monitoring animal welfare.

## 5. Conclusions

Over the last 5 to 10 years, the definition of animal welfare has evolved to encompass the concept that animals should experience a “life worth living”. Accordingly, the next generation of biomarkers for evaluating animal welfare needs to reflect a positive welfare state and a positive mental status. Modern assessment methods of animal welfare need to match current and future welfare standards. Our findings describe for the first time in a cattle model a complete picture of the BDNF and the kynurenine pathway involved in the chronic stress induced by tie-stall housing and suggest that such systems may modulate emotional states in dairy cows.

The biomarkers we investigated will need to be validated in larger study samples. Our findings support their potential inclusion in the welfare evaluation protocols in current use. The biomarkers could improve the welfare assessment of dairy cows and other farmed animals and provide a holistic view of the health state of animals in intensive farming.

## Figures and Tables

**Table 1:** General data of the dairy cattle.

Farm	Sample ID	Breed	Sex	Age (mths) at Blood Draw	Lactation State	Age (mths) at Culling	Brain Collected
A	A1	FRS	F	69	Full	73	Yes
	A2	FRS	F	94	Full	123	Yes
	A3	FRS	F	49	Full	95	Yes
	A4	FRS	F	61	Full	82	Yes
	A5	FRS	F	50	Full	n.a.	No
	A6	FRS	F	62	Full	n.a.	No
	A7	FRS	F	128	Full	n.a.	No
	A8	FRS	F	119	Full	n.a.	No
	A9	FRS	F	72	Full	n.a.	No
	A10	FRS	F	59	Full	n.a.	No
	A11	FRS	F	67	Full	n.a.	No
	A12	FRS	F	33	Full	n.a.	No
	A13	FRS	F	82	Full	n.a	No
B	B1	MTT	F	121	Full	n.a.	No
	B2	FRS	F	126	Full	138	Yes
	B3	MTT	F	118	Full	n.a.	No
	B4	FRS	F	108	Full	n.a.	No
	B5	FRS	F	108	Full	n.a.	No
	B6	FRS	F	91	Full	n.a.	No
	B7	FRS	F	80	Full	n.a.	No
	B8	FRS	F	75	Full	n.a.	No
	B9	FRS	F	75	Full	n.a.	No
	B10	FRS	F	72	Full	85	Yes
	B11	FRS	F	56	Full	n.a.	No
	B12	FRS	F	66	Full	n.a.	No
	B13	FRS	F	66	Full	75	Yes
C	C1	FRS	F	89	Full	97	Yes
	C2	FRS	F	50	Full	n.a.	No
	C3	FRS	F	63	Full	n.a.	No
	C4	FRS	F	93	Full	96	Yes
	C5	FRS	F	109	Full	n.a.	No
	C6	FRS	F	51	Full	n.a.	No
	C7	FRS	F	86	Full	n.a.	No
	C8	FRS	F	61	Full	73	Yes
	C9	FRS	F	37	Full	n.a.	No
	C10	FRS	F	117	Full	n.a.	No
	C11	FRS	F	103	Full	106	Yes
	C12	FRS	F	92	Full	125	Yes
	C13	FRS	F	142	Full	n.a.	No
	C14	FRS	F	80	Full	n.a.	No
	C15	FRS	F	61	Full	n.a.	No

FRS denotes Holstein Friesian; MTT, crossbred; mths, months; n.a., not available.

**Table 2:** Total animal welfare scores and section scores in the on-farm animal welfare assessment. Section and total welfare scores are expressed in percentages from 0 to 100, where 0 denotes a poor level or poor animal welfare status and 100 denotes an excellent section level or optimal animal welfare status.

Group or Farm	Farm Type	Milk Production	Total Animal Welfare Score (%)	Section Score (%)	
Farm A	Free-stall	≤40 kg/cow per day	65.5	^1^ A	52.8
				^2^ B	56.7
				^3^ C	76.1
Farm B	Tie-stall	≤12.5 kg/cow per day	48.9	A	43.0
				B	43.7
				C	54.5
Farm C	Free-stall	>40 kg/cow per day	70.5	A	84.7
				B	63.5
				C	67.4

^1^ Section A—farm management and staff training; ^2^ Section B—housing and equipment; ^3^ Section C—animal-based measures.

**Table 3:** Blood parameters in lactating cows: mean and confidence intervals (>95%).

Characteristic	Farm A		Farm B		Farm C		
	Mean	CI	Mean	CI	Mean	CI	Reference Value
WBCs ^1^ (m/mm^3^)	8.4	6.5–16.8	7.5	6.31–9.27	8.0	7.0–8.9	4–12 m/mm^3^
LYM%	35.6	31.8–42.1	38.4	34.9–41.7	37.8	34.8–41.2	45–75%
MONO%	4.2	3.8–4.8	3.9	3.6–4.2	4.6	4.0–5.2	1–5%
NEU%	54.5	49.5–60.1	54.4	51.9–57.5	54.4	51.5–57.8	15–47%
EOS%	2.7	1.9–3.4	2.6	2.02–3.32	3.4	2.5–4.7	2–20%
BAS%	0.4	0.33–0.6	0.4	0.3–0.5	0.4	0.31–0.54	-
RBCs (m/mm^3^)	6.3	5.83–6.76	6.4	6.0–6.8	7.1	6.7–7.4	6–11 m/mm^3^
MCV (fL)	48.6	46.35–51.62	42.1	39.2–46.4	49.7	47.6–52.1	40–60 fL
HCT%	30.0	27.84–31.92	26.6	24.6–28.7	35.4	33.7–37.2	25–50%
MCH (pg)	15.4	14.22–16.65	13.0	12.2–14.2	16.7	15.6–17.7	11–17 pg
MCHC (g/dL)	31.8	30.6–32.9	31.2	30.7–31.8	33.4	32.5–34.3	30–40 g/dL
RDW	13.2	12.8–13.6	13.8	13.03–14.6	13.0	12.56–13.36	8–12
HB (g/dL)	9.5	8.90–10.19	8.25	7.6–8.9	12.0	11.24–12.57	8–15 g/dL
PLTs (m/mm^3^)	238.0	165.7–408.4	166.8	144.7–211.3	193.3	165.1–226.3	100–800 m/mm^3^
MPV (fL)	8.1	7.7–8.5	8.0	7.32–8.82	8.1	7.77–8.44	3–8 fL
PCT%	0.19	0.13–0.34	0.14	0.12–0.17	0.15	0.13–0.17	-

^1^ White blood cells (WBCs), percentage of lymphocytes (LYM%), percentage of monocytes (MONO%), percentage of eosinophils (EOS%), percentage of basophils (BAS%), erythrocytes (RBCs), mean corpuscular volume (MCV), hematocrit (HCT), mean corpuscular hemoglobin (MCH), mean corpuscular hemoglobin concentration (MCHC), hemoglobin (HB), red cell volume distribution (RDW), platelets (PLTs), mean platelet volume (MPV), thrombocytocrit (PCT%).

**Table 4:** Blood biochemical parameters in lactating cows: mean and confidence intervals (>95%).

Characteristic	Farm A		Farm B			Farm C	
	Mean	CI	Mean	CI	Mean	CI	Reference Value
ALP ^1^ (UI/L)	32.4	14.7–59.6	32.1	22.2–49.8	32.1	24.5–49.9	100–488 UI/L
T-Chol (mg/dL)	112.5	92.6–126.2	109.3	99.0–128.3	111.4	90.5–132.0	80–120 mg/dL
CREA (mg/dL)	1	0.9–1.1	1	0.9–1.1	1	0.9–1.1	1–2 mg/dL
AST (UI/L)	78.8	70.6–88.7	78.7	67.9–91.4	78.7	71.3–86.8	36–80 UI/L
ALT (UI/L)	26.2	22.4–30.5	26.3	22.9–30.8	26.2	24.7–29.5	20–60 UI/L
TG (mg/dL)	27.7	18.3–43.8	27.7	22.1–34.1	27.5	24.1–34.6	0–14 mg/dL
UR (mg/dL)	76.3	56.2–114.2	76.2	53.4–102.8	76.3	59.6–95.0	20–30 mg/dL
Lysozyme (µg/mL)	1.6	1.2–1.8	2.1	1.3–1.8	1.5	1.3–1.8	1–3 ug/mL
Bactericidal activity (%)	82.4	74.9–88.7	76.8	71.5–82.6	72.2	61.7–79.5	>90%
CRP (mg/L)	6.1	4.1–10.6	6.1	3.9–10.7	6.1	4.8–9.4	[31]

^1^ Alkaline phosphatase (ALP), total cholesterol (T-Chol), creatinine (CREA), aspartate aminotransferase (AST), alanine aminotransferase (ALT), triglyceride (TG), urea (UR), C-reactive protein (CRP).

**Table 5:** Immunological parameters in lactating cows: estimated mean ± standard deviation and range.

Item	Farm A		Farm B		Farm C		KW *p*-Value
	Mean ± SD	Range	Mean ± SD	Range	Mean ± SD	Range	
INF γ	1.8 ± 2.5	0.03–7.6	1.3 ± 1.2	0.07–3.7	0.7 ± 0.5	0.03–1.6	NS
IL-1 β	13.5 ± 16.3	4.0–63.2	12.2 ± 16.2	1.06–63.2	8.6 ± 3.8	11.4–13.2	NS
IL-6	361.1 ± 434.4	106.3–1685.4	325.6 ± 432.3	28.5–1685.4	228.0± 100	28.3–464.8	NS
IL-36Ra	243.2 ± 88.25 ^a^	134.1–375.6	393.7 ± 179.3 ^a^	212.6–916.7	247.6 ± 184.5 ^A^	100.5–786.2	0.004 *
IL-8	358.8 ± 256.1	85.8–853.9	501.3 ± 260.3	98.6–845.9	545.4 ± 329.8	171.6–1185.9	NS
IL-10	249.8 ± 328.3 ^A^	40.5–909.4	149.0 ± 308.3 ^A^	3.7–1165.7	545.4 ± 329.7 ^A^	171.6–1185.9	0.0004 *
IP-10	750.0 ± 277.6	397.2–1315.1	689.2 ± 317.2	331.6–1449.7	553.8 ± 239.2	190.3–1040.2	NS
MCP-1	382.9 ± 245.6	148.9–855.0	236.9 ± 203.6	126.2–902.8	197.1 ± 251.9	92.7–778.0	NS
MIP-1 α	546.5 ± 486.7	150.3–1604.9	484.2 ± 472.3	165.7–1842.5	491.1 ± 351.2	155.1–1075.2	NS
MIP-1 β	183.7 ± 275.4	22.0–737.6	28.2 ± 20.5	5.3–64.6	341.5 ± 541.2	7.9–1459.9	NS
TNF α	2373.0 ± 3760.1	6.5–0.1	2072.4 ± 4487.1	94.6–0.9	2203.2 ± 2347.3	112.1–6061.9	NS
VEGF-A	98.3 ± 85.8	28.2–261.5	58.9 ± 57.8	21.6–235.7	56.5 ± 46.8	16.7–170.6	NS

* Note: Measurements with common superscript letters in the same row differ at *p* ≤ 0.05 for lowercase and *p* ≤ 0.01 for uppercase. All measurements are expressed as picogram/mL. NS denotes non-significant, *p* > 0.05.

**Table 6:** Comparison of metabolomic parameters between Farms A, B, and C according to the non-parametric local–linear and the local–constant kernel regression model.

Item	Farm B vs. A		Farm C vs. A		Farm C vs. B	
	Contrast	*p*-Value	Contrast	*p*-Value	Contrast	*p*-Value
* PCaeC34:0	1.2718	0.0188	−0.7547	0.0348	−2.0265	0.0001
PcaeC34:1	5.1899	0.0239	−5.0076	0.0019	−10.1975	<0.0001
PcaeC36:1	10.1473	0.0001	−4.1098	0.0007	−14.2571	<0.0001
PcaeC38:1	1.2871	<0.0001	−0.4570	0.0180	−1.7441	<0.0001
PcaeC38:6	−1.2760	<0.0001	1.1440	0.0339	2.4200	<0.0001
lysoPCaC18:2	−12.7029	<0.0001	5.8931	0.1150	18.5960	<0.0001
lysoPCaC20:3	−0.7781	0.0013	0.3841	0.1808	1.1622	0.0001
lysoPCaC24:0	0.0614	0.1707	−0.0366	0.1782	−0.0981	0.0206
PcaaC30:2	0.0549	0.5215	−0.1869	0.0214	−0.2418	0.0054
PcaaC32:2	1.8085	0.1393	−2.9221	0.0051	−4.7307	0.0004
PcaaC32:3	−16.2434	<0.0001	−4.4606	0.2655	11.7827	0.0016
PcaaC34:4	−3.5402	<0.0001	−0.0565	0.9564	3.4838	0.0008
PcaaC36:2	−208.8599	<0.0001	27.3927	0.5932	236.2526	<0.0001
PcaaC36:3	−39.6146	0.0001	23.3819	0.1492	62.9965	0.0002
PcaaC36:4	−11.2410	0.0002	7.5451	0.0868	18.7860	<0.0001
PcaaC38:1	2.4087	0.0001	−0.0081	0.9764	−2.4168	0.0001
PcaaC38:3	−18.2647	0.0051	2.4244	0.7168	20.6891	0.0114
PcaaC42:5	0.5513	0.0044	0.3411	0.0037	−0.2102	0.2611
PcaaC42:6	0.2511	0.0018	0.0118	0.8201	−0.2393	0.0025
PcaeC36:0	0.8041	0.0067	−0.0577	0.6736	−0.8618	0.0063
PcaaC34:1	7.2690	0.5835	−13.0623	0.2960	−20.3313	0.0384
PcaaC34:2	−124.3028	<0.0001	19.5469	0.4493	143.8498	<0.0001
PcaaC24:0	0.0541	0.2098	−0.0243	0.4353	−0.0784	0.0500
** SMC16:0	−34.7889	0.0034	−9.8360	0.3842	24.9530	0.0624
SMC16:1	−3.6809	0.0019	−0.0886	0.9402	3.5923	0.0210
SMC18:1	−3.2701	<0.0001	0.7133	0.4747	3.9834	0.0001
SM(OH)C22:1	−10.2340	0.0010	−4.7224	0.0661	5.5116	0.0233
SM(OH)C22:2	2.9793	0.0504	−1.7553	0.0238	−4.7346	0.0039
Tryptophan	−7.7893	0.0387	10.0719	0.0189	17.8612	0.0002
Histidine	−34.6812	<0.0001	9.1781	0.2986	43.8592	<0.0001
Isoleucine	−38.0402	0.0138	18.6727	0.2427	56.7129	<0.0001
KYN/TRP	0.1694	0.0112	−0.0282	0.2913	−0.1976	0.0028
Kynurenine	3.8764	0.0067	−0.0424	0.9688	−3.9187	0.0055
Taurine	−45.6090	<0.0001	15.2728	0.1333	60.8818	<0.0001
Threonine	−27.2245	0.0426	18.8465	0.1847	46.0710	0.0003
Valine	−103.9259	0.0001	24.7220	0.3731	128.6479	<0.0001
Proline	−22.9941	0.0125	16.2568	0.2103	39.2509	0.0021
Putrescine	−0.1231	0.0018	−0.0326	0.4132	0.0905	0.0009
Putrescine/Ornithine	−0.0014	0.0249	−0.0004	0.4427	0.0009	0.0487
alpha-AAA	−0.9372	0.0008	0.2277	0.5153	1.1648	0.0003

* PC denotes phosphatidylcholine diacyl; ** SM, sphingomyelin.

**Table 7:** Descriptive statistics of plasma BDNF concentration by farm.

Farm	n	min	maxi	mean	sd	p25	p50	p75
A	13	106.2	2896.6	583.4	755.6	221.2	374.8	496.3
B	13	111.5	5824.3	1281.63	1763.8	440.9	542.8	926.3
C	15	41.6	979.7	233.93	253.7	61.8	146.9	237.8

## Data Availability

The data presented in this study are available within the article and the Supplementary Materials.

## Supplementary Materials

The following supporting information can be downloaded at: https://www.mdpi.com/article/10.3390/ani13071167/s1, Figure S1: Cattle age distribution in farm A, B and C; Figures S2 and S3: Western Blot analysis on hippocampus to measure the expression levels of mBDNF and pro-BDNF; Figure S4: Correlation between cortical and thalamic IDO1 levels; Table S1: Concentrations of plasma metabolites; Table S2: VIP scores.
