# Supplementary material for: Brain-Derived Neurotrophic Factor, Kynurenine Pathway, and Lipid-Profiling Alterations as Potential Animal Welfare Indicators in Dairy Cattle
Source: Animals (Basel). 2023 Mar 25;13(7):1167. doi: 10.3390/ani13071167 (PMC10093196; doi:10.3390/ani13071167)
Supplement: Supplementary file 1 [file animals-13-01167-s001.zip › Supplementary Figures.pptx]

## Slide 1
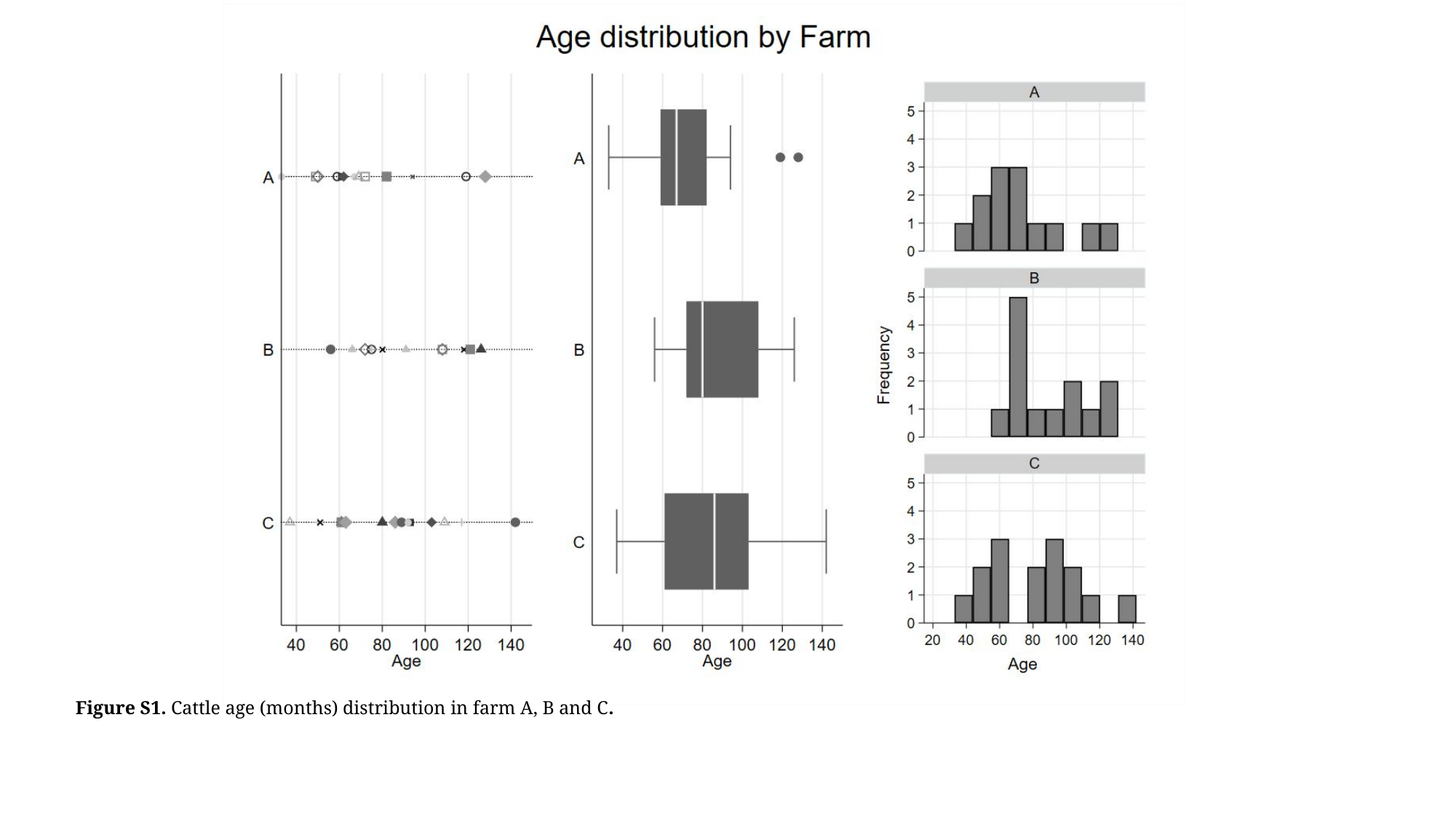

Figure S1. Cattle age (months) distribution in farm A, B and C.

## Slide 2
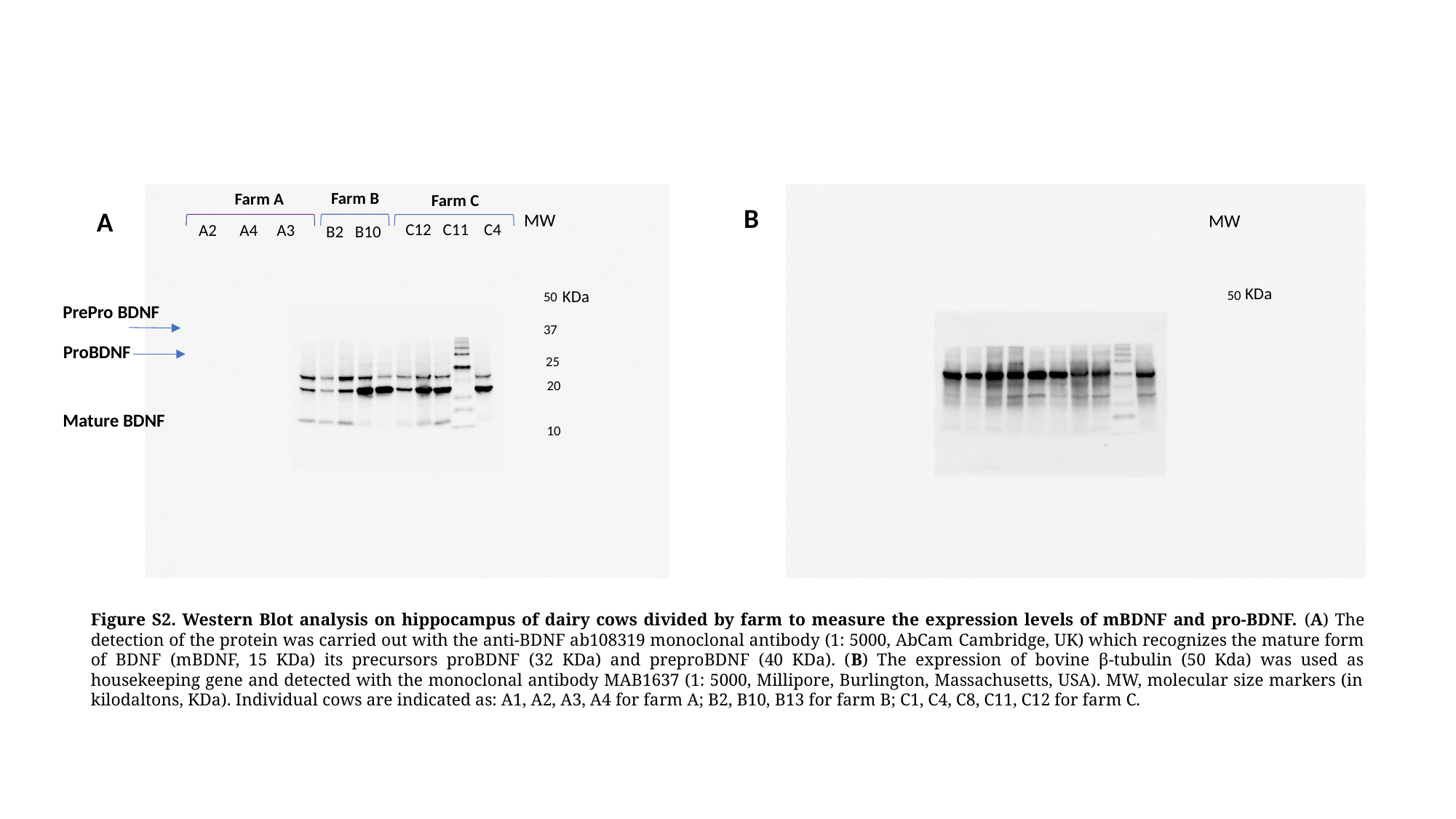

Farm B
Farm A
Farm C
B
A
MW
MW
C12 C11 C4
A2 A4 A3
B2 B10
KDa
KDa
50
50
PrePro BDNF
37
ProBDNF
25
20
Mature BDNF
10
Figure S2. Western Blot analysis on hippocampus of dairy cows divided by farm to measure the expression levels of mBDNF and pro-BDNF. (A) The detection of the protein was carried out with the anti-BDNF ab108319 monoclonal antibody (1: 5000, AbCam Cambridge, UK) which recognizes the mature form of BDNF (mBDNF, 15 KDa) its precursors proBDNF (32 KDa) and preproBDNF (40 KDa). (B) The expression of bovine β-tubulin (50 Kda) was used as housekeeping gene and detected with the monoclonal antibody MAB1637 (1: 5000, Millipore, Burlington, Massachusetts, USA). MW, molecular size markers (in kilodaltons, KDa). Individual cows are indicated as: A1, A2, A3, A4 for farm A; B2, B10, B13 for farm B; C1, C4, C8, C11, C12 for farm C.

## Slide 3
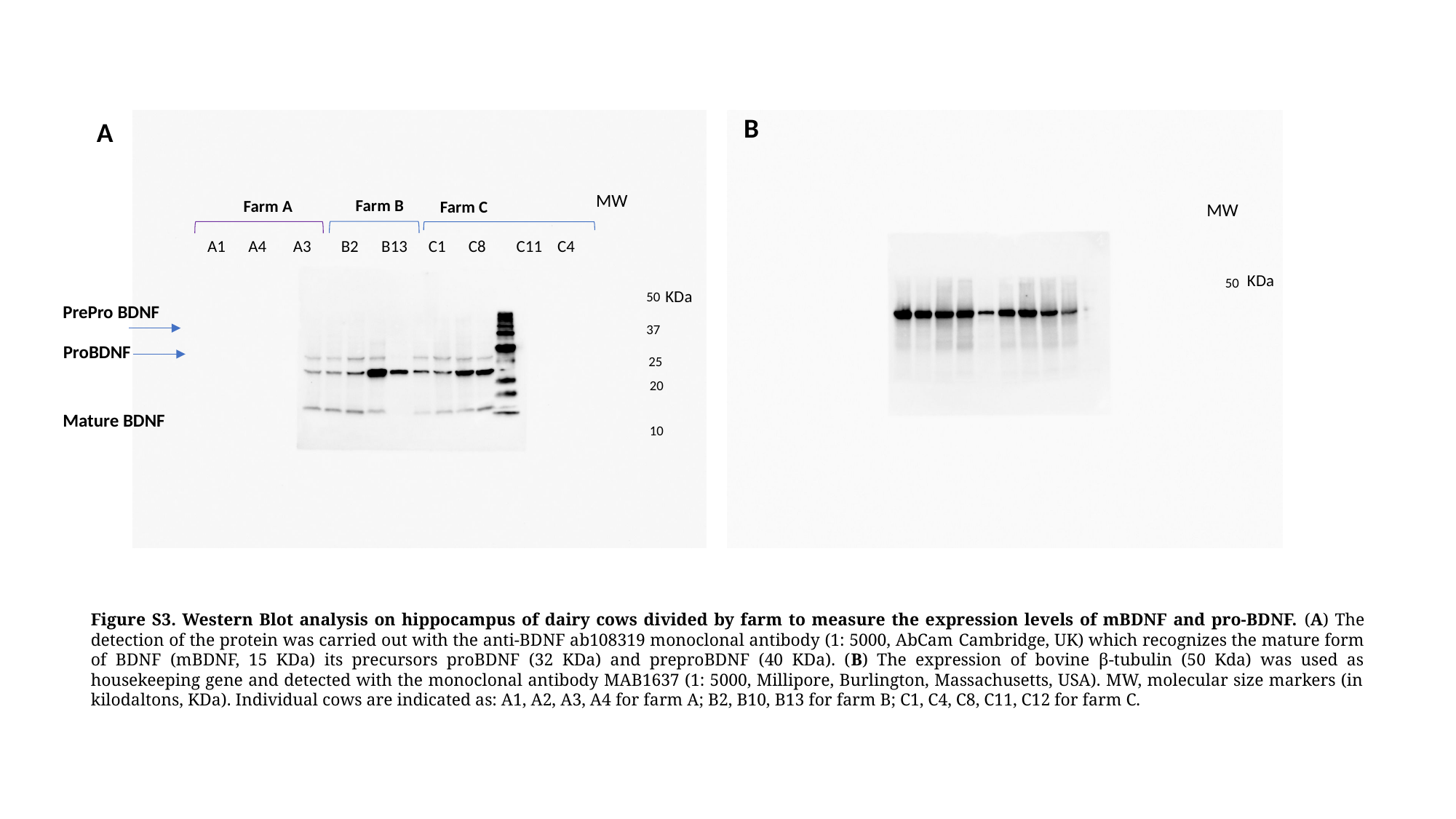

B
A
MW
Farm B
Farm A
Farm C
MW
 A1 A4 A3
 B2 B13
C1 C8 C11 C4
KDa
50
KDa
50
PrePro BDNF
37
ProBDNF
25
20
Mature BDNF
10
Figure S3. Western Blot analysis on hippocampus of dairy cows divided by farm to measure the expression levels of mBDNF and pro-BDNF. (A) The detection of the protein was carried out with the anti-BDNF ab108319 monoclonal antibody (1: 5000, AbCam Cambridge, UK) which recognizes the mature form of BDNF (mBDNF, 15 KDa) its precursors proBDNF (32 KDa) and preproBDNF (40 KDa). (B) The expression of bovine β-tubulin (50 Kda) was used as housekeeping gene and detected with the monoclonal antibody MAB1637 (1: 5000, Millipore, Burlington, Massachusetts, USA). MW, molecular size markers (in kilodaltons, KDa). Individual cows are indicated as: A1, A2, A3, A4 for farm A; B2, B10, B13 for farm B; C1, C4, C8, C11, C12 for farm C.

## Slide 4
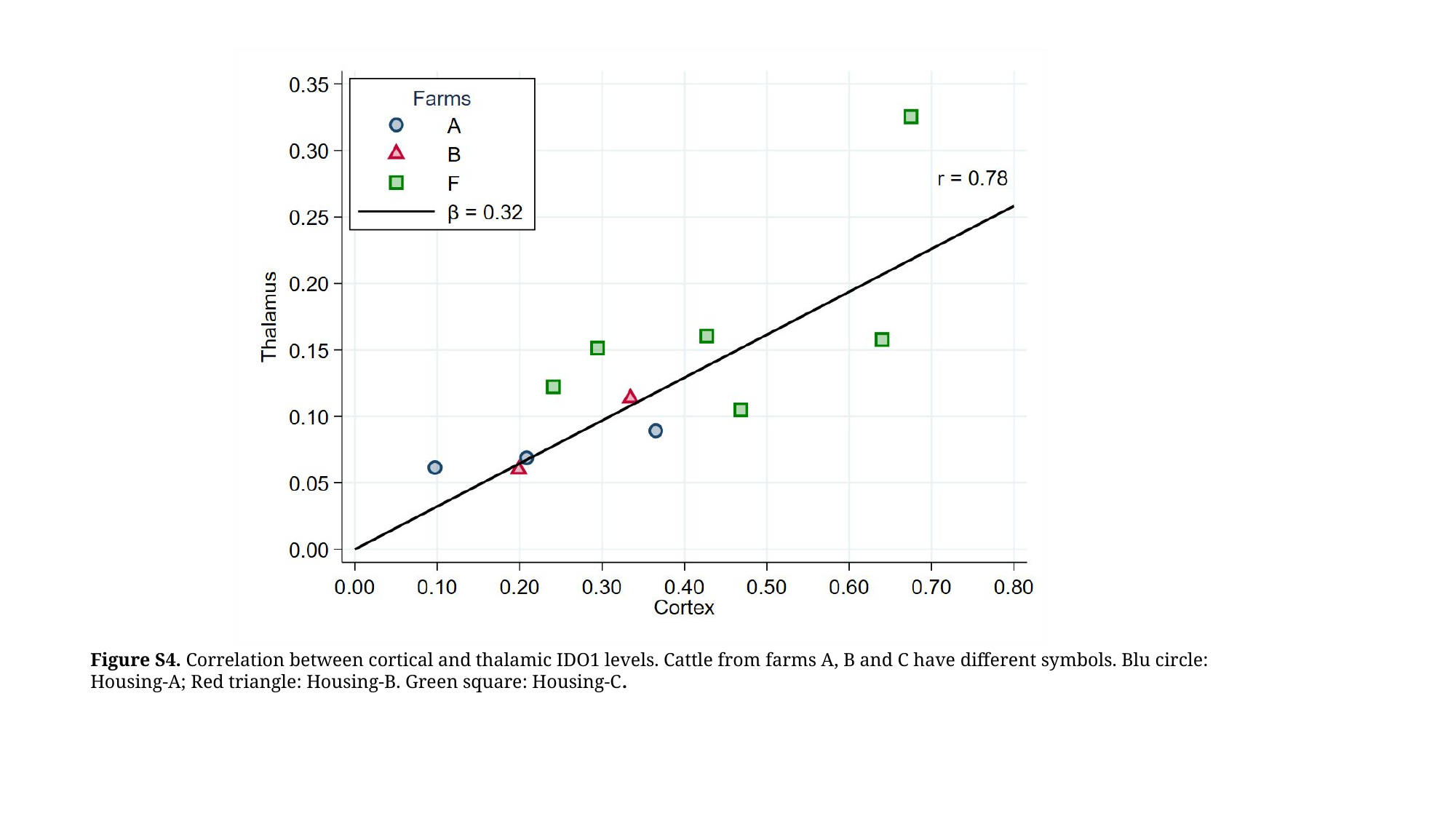

Figure S4. Correlation between cortical and thalamic IDO1 levels. Cattle from farms A, B and C have different symbols. Blu circle: Housing-A; Red triangle: Housing-B. Green square: Housing-C.
